# Hippocampal but Not Serum Cytokine Levels Are Altered by Traffic-Related Air Pollution in TgF344-AD and Wildtype Fischer 344 Rats in a Sex- and Age-Dependent Manner

**DOI:** 10.3389/fncel.2022.861733

**Published:** 2022-04-22

**Authors:** Kelley T. Patten, Anthony E. Valenzuela, Christopher Wallis, Danielle J. Harvey, Keith J. Bein, Anthony S. Wexler, Fredric A. Gorin, Pamela J. Lein

**Affiliations:** ^1^Department of Molecular Biosciences, School of Veterinary Medicine, University of California, Davis, Davis, CA, United States; ^2^Air Quality Research Center, University of California, Davis, Davis, CA, United States; ^3^Department of Public Health Sciences, School of Medicine, University of California, Davis, Davis, CA, United States; ^4^Center for Health and the Environment, University of California, Davis, Davis, CA, United States; ^5^Mechanical and Aerospace Engineering, Civil and Environmental Engineering, College of Engineering, University of California, Davis, Davis, CA, United States; ^6^Land, Air and Water Resources, College of Agricultural and Environmental Sciences, University of California, Davis, Davis, CA, United States; ^7^Department of Neurology, Davis School of Medicine, University of California, Sacramento, Sacramento, CA, United States

**Keywords:** air pollution, Alzheimer’s disease, cytokines, IL-1β, microglia, neuroinflammation

## Abstract

Epidemiological studies have demonstrated that air pollution is a significant risk factor for age-related dementia, including Alzheimer’s disease (AD). It has been posited that traffic-related air pollution (TRAP) promotes AD neuropathology by exacerbating neuroinflammation. To test this hypothesis, serum and hippocampal cytokines were quantified in male and female TgF344-AD rats and wildtype (WT) Fischer 344 littermates exposed to TRAP or filtered air (FA) from 1 to 15 months of age. Luminex™ rat 23-cytokine panel assays were used to measure the levels of hippocampal and serum cytokines in 3-, 6-, 10-, and 15-month-old rats (corresponding to 2, 5, 9, and 14 months of exposure, respectively). Age had a pronounced effect on both serum and hippocampal cytokines; however, age-related changes in hippocampus were not mirrored in the serum and vice versa. Age-related changes in serum cytokine levels were not influenced by sex, genotype, or TRAP exposure. However, in the hippocampus, in 3-month-old TgF344-AD and WT animals, TRAP increased IL-1ß in females while increasing TNF ɑin males. In 6-month-old animals, TRAP increased hippocampal levels of M-CSF in TgF344-AD and WT females but had no significant effect in males. At 10 and 15 months of age, there were minimal effects of TRAP, genotype or sex on hippocampal cytokines. These observations demonstrate that TRAP triggers an early inflammatory response in the hippocampus that differs with sex and age and is not reflected in the serum cytokine profile. The relationship of TRAP effects on cytokines to disease progression remains to be determined.

## Introduction

Alzheimer’s disease (AD) is the most prevalent cause of age-related dementia. At present, there are limited therapeutic options for preventing or even slowing the progression of this disease, thus, considerable efforts are being made to understand modifiable risk factors, such as environmental exposures (Eid et al., 2019; Mir et al., 2020). One environmental factor strongly associated with increased risk of AD is chronic exposure to traffic-related air pollution (TRAP) (Peters et al., 2019; Fu and Yung, 2020). Recent studies suggest that TRAP interacts with non-modifiable risk factors for AD, such as genetic susceptibilities and age, to influence individual AD risk. For example, women > 65 years with the APOE-e4 risk allele for AD who were exposed to high levels of particulate matter 2.5 (PM_2_._5_) showed increased risk of cognitive decline and dementia compared to APOE-ε4 women exposed to low PM_2_._5_ levels (Cacciottolo et al., 2017). However, how environmental risk factors, aging, and genetic susceptibilities interact to influence AD risk remains largely unknown.

Neuroinflammation is associated with TRAP exposure, aging, and AD pathogenesis (Calderon-Garciduenas et al., 2016; Guzman-Martinez et al., 2019). Several studies have shown increased levels of pro-inflammatory cytokines such as IL-6 and TNFɑin the serum and cerebrospinal fluid (CSF) of aged individuals compared to young controls (Michaud et al., 2013a). Likewise, a growing list of AD genetic risk factors have been linked to neuroinflammation (Kloske and Wilcock, 2020). For example, APOE-e4 may play a role in nitric oxide regulation in the brain (Vitek et al., 2009), while mutations or single-nucleotide polymorphisms in innate immune genes such as *TREM2* (triggering receptor expressed on myeloid cells 2) (Liddelow et al., 2017), *CR1* (complement receptor 1) (Efthymiou and Goate, 2017), and *CD33* (Karch and Goate, 2015) have been associated with altered microglial function in the context of AD. Many of these innate immune genes are also expressed in peripheral immune cells, such as leukocytes, monocytes, and macrophages (Forabosco et al., 2013; Guerreiro et al., 2013; Jonsson et al., 2013), and may contribute to altered peripheral inflammation in AD (Cao and Zheng, 2018). Finally, cytokines such as TNFɑ, IL-1β, and IL-6 are increased in the brains and serum of experimental animals and humans exposed to air pollution (Mumaw et al., 2016; Jayaraj et al., 2017).

Immune activity in the CNS is controlled in part by cytokines and chemokines, and their differential expression in the brains of AD patients is an active area of research. Cytokines and chemokines are paracrine and autocrine signaling molecules that are crucial in sensing and responding to damage in the brain. Increased levels of these cytokines are often observed in neurodegenerative processes. For example, injured neurons in the brain release cytokines such as IL-1b and tumor-necrosis factor alpha (TNFɑ) (Griffin and Barger, 2010; Stamouli and Politis, 2016), which can lead to neuronal apoptosis or the activation of local glial cells. The release of chemokines such as C-C motif ligand 2 (CCL2; also known as monocyte chemoattractant protein 1 or MCP-1) from glia can recruit monocytes or microglia to sites of damage in the brain (Platten et al., 2003; Kim et al., 2014). In AD, Ab peptides and plaques are known to activate surrounding microglia, leading to increased levels of proinflammatory cytokines such as TNFɑand IL-6 in the area adjacent to the plaque (Rubio-Perez and Morillas-Ruiz, 2012; Wang et al., 2015). Conversely, anti-inflammatory cytokines such as IL-4 and IL-10 can dampen microglial activation and thereby reduce the damaging effects of pro-inflammatory cytokines (Gadani et al., 2012), or they may decrease the ability of microglia to phagocytose Ab (Guillot-Sestier et al., 2015). These examples illustrate the complex effects of cytokines in AD, which have been previously reviewed (Cacquevel et al., 2004; Angelopoulos et al., 2008; Reale et al., 2008; Lee et al., 2009; Rubio-Perez and Morillas-Ruiz, 2012; Nagae et al., 2016; Su et al., 2016).

Cytokines and chemokines in the serum are of interest as biomarkers of disease progression in AD patients. Although cytokine levels in CSF are often highly correlated with levels in brain tissue (Mitrasinovic et al., 2003; Choi et al., 2008; Olson and Humpel, 2010), obtaining CSF requires a lumbar puncture and therefore is not routinely evaluated in AD patients. Consequently, there is great interest in identifying prognostic AD biomarkers in blood. Given the strong role of inflammation in AD, numerous studies have measured cytokines in the serum of patients with mild cognitive impairment or AD. However, the results of these studies have been inconsistent. For example, TNFɑserum levels, which are known to be elevated in conditions such as depression, have been evaluated in several AD studies with contradictory results (Zheng et al., 2016). Several studies have shown that serum TNFɑlevels were elevated in AD patients with advanced disease, compared to those with mild disease (Morimoto et al., 2011). Additional studies have found no difference in TNFɑconcentrations between AD patients and controls (Guerreiro et al., 2007; Khemka et al., 2014; Lai et al., 2017). Similar contradictory findings have been reported for IL-6 and transforming-growth factor beta (TGF-b) (Angelopoulos et al., 2008; Khemka et al., 2014; Lai et al., 2017).

An objective of this study was to identify potential biomarkers that would reflect interactions between TRAP and ongoing neurodegeneration in a rat model of AD. We analyzed samples taken from a larger study (Patten et al., 2021) characterizing the effects of chronic traffic-related air pollution (TRAP) exposure on expression of AD phenotypes in wildtype (WT) vs. transgenic rats that expressed human AD risk genes (TgF344-AD rats). In the larger study, we demonstrated that exposure to TRAP accelerated amyloid plaque deposition, increased hyperphosphorylated tau levels, promoted neuronal cell loss, and caused cognitive deficits in an age, genotype and sex-dependent manner (Patten et al., 2021). We also observed that TRAP increased microglial cell activation, but not astrogliosis in both male and female WT and TgF344-AD rats (Patten et al., 2021). We measured the profiles of cytokines, chemokines, and growth factors in serum and hippocampal tissue as affected by age, TRAP exposure, and AD genetic susceptibility, and whether there were any interactions between these risk factors.

## Materials and Methods

### Animals

All procedures involving animals were conducted in accordance with the NIH Guide for the Care and Use of Laboratory Animals and were approved by the University of California Davis Animal Care and Use Committee. Protocols conformed to the ARRIVE guidelines (Kilkenny et al., 2010). Animal facilities were maintained under controlled environmental conditions (20–26°C, 12:12 light dark cycle) with food and tap water provided *ad libitum*. Rats were fed a Global 18% Protein Rodent Diet (Tekland) throughout the study.

The animals used to generate the data reported here were part of a larger study designed to evaluate the effects of TRAP on cardiovascular and AD phenotypes and have been described previously (Edwards et al., 2020; Patten et al., 2021). Briefly, male TgF344-AD rats (Cohen et al., 2013) that express the human AD risk genes APPswe (Swedish mutation) and presenilin-1 Δ exon 9 (PS1ΔE9 mutation) were obtained from Emory University and housed in vivaria on the University of California, Davis campus. Hemizygous male TgF344-AD rats were paired with female wildtype CDF344 (WT) rats from Charles River Laboratories (Shrewsbury, MA, United States) to generate 60 litters of hemizygous and WT males and females. Animals were genotyped at postnatal day (PND) 8, weaned at PND 21, and randomly assigned to TRAP- or FA-exposure groups. At ∼PND 28, animals were transported to the tunnel exposure facility (described below). Multiple cages with open-wire lids were placed inside each exposure chamber, and thus animals were exposed to whole-body FA or TRAP. The longest exposure for animals was 14 months from September 2017 to November 2018. Cytokine analyses were performed on 4–5 animals per group.

### Tunnel Exposure Facility

The tunnel exposure facility, which was inspected and approved by the UC Davis Institutional Animal Care and Use Committee, has been previously described (Edwards et al., 2020; Patten et al., 2020, 2021). Briefly, a vivarium was built within a mobile facility adjacent to a major highway tunnel, and air from the tunnel was delivered unchanged in real time to exposure chambers inside the vivarium. Both light- and heavy-duty vehicular traffic contributed to vehicular emissions within the tunnel. Control animals housed in the same vivarium adjacent to the tunnel were exposed to ambient air captured immediately outside the facility that was sequentially filtered through: (1) A coarse filter to remove large debris; (2) activated carbon to remove volatile organic compounds; (3) barium oxide-based catalytic converters to remove nitrogen oxides; and (4) ultra-high efficiency filters to remove coarse, fine, and ultrafine particulate matter (PM) before being delivered to the FA exposure chamber. Briefly, mean 24 h PM_2_._5_ levels were 0.25 ± 0.11 μg/m^3^ in FA and 15.6 ± 3.7 μg/m^3^ in TRAP, which is well below the United States 24-h PM_2_._5_ National Ambient Air Quality Standard of 35 μg/m^3^ (Patten et al., 2021).

### Tissue Collection and Processing

After 2, 5, 9, or 14 months of exposure, corresponding to animals aged 3, 6, 10, and 15 months, respectively), a subset of animals was transported back to the University of California, Davis campus. After 18 h recovery, animals were euthanized as previously described (Patten et al., 2021), and hippocampal tissue and serum were collected. Briefly, rats were deeply anesthetized using 4% isoflurane (Southmedic Inc., Barrie ON) in a 2:1 mixture of medical grade air and medical grade oxygen delivered *via* inhalation at a rate of 1.5 L/min. The chest cavity was opened and whole blood was collected from the heart *via* cardiac puncture into serum separator tubes (Becton-Dickinson, East Rutherford, NJ). Tubes stood for 30 min at room temperature before centrifugation at 1,500 × g for 10 min. Serum was pipetted into individual microcentrifuge tubes and immediately frozen and stored at –80 C. Immediately after collecting blood, rats were transcardially perfused with 100 ml of cold 0.1 M phosphate buffered saline (PBS; pH 7.2, 137 mM NaCl, 10 mM sodium phosphate dibasic, 1.8 mM potassium phosphate monobasic) at a rate of 15 ml/min using a Masterflex peristaltic pump (Cole Parmer, Vernon Hills, IL). The brain was removed and bisected sagittally using a stainless-steel rat brain matrix (Zivic Instruments, Pittsburgh, PA). The left hemisphere hippocampus was removed by microdissection on ice, snap-frozen in individual tubes, and immediately stored at –80°C until use. Other tissues were collected for additional immunohistochemical and biochemical analyses that were previously described (Edwards et al., 2020; Patten et al., 2021).

### Cytokine and Chemokine Analyses

Both serum and hippocampal cytokines and chemokines were quantified using Bio-Plex Pro™ 23-plex cytokine assays (Bio-Rad, Hercules, CA) of the same lot. Kit analytes included the following: interleukin-1beta (IL-1β), interleukin-18 (IL-18), interleukin-1alpha (IL-1ɑ), tumor necrosis factor-alpha (TNFɑ), interleukin-6 (IL-6), interleukin-12 (IL-12), which is also known as p70, interkeukin-17 (IL-17), interleukin-7 (IL-7), interleukin-2 (IL-2), interferon-gamma (IFN_ɣ_), interleukin-4 (IL-4), interleukin-5 (IL-5), interleukin-10 (IL-10), interleukin-13 (IL-13), granulocyte colony-stimulating factor (G-CSF), granulocyte-macrophage colony-stimulating factor (GM-CSF), macrophage colony-stimulating factor (M-CSF), C-X-C motif ligand 1/keratinocyte chemoattractant (CXCL1/KC), C-C motif ligand 2/monocyte chemoattractant protein 1 (CCL2/MCP-1), C-C motif ligand 3/macrophage inflammatory protein-1 alpha (CCL3/MIP-1ɑ), C-C motif ligand 5/regulated upon activation, normal T cells, expressed, and secreted (CCL5/RANTES), C-C motif ligand 20/macrophage inflammatory protein-3 alpha (CCL20/MIP3ɑ), and vascular endothelial growth factor (VEGF).

Assays were performed in accordance with the manufacturer’s instructions, with modifications as follows: For hippocampal tissue lysates, 300 mg of frozen hippocampal tissue per animals was homogenized with a hand-held VirSonic ultrasonic cell disruptor 100 (VirTis, Gardiner, NY), using 5–10 pulses of 1 s per sample. Tissue samples were homogenized in 1x cell lysis buffer (BioRad) supplemented with cOmplete™ protease inhibitor cocktail (Sigma Aldrich, St. Louis, MO). After homogenization, samples were frozen for 15 min on dry ice, thawed, and then centrifuged at 4,500 rcf for 4 min at 4°C. Levels of total protein in the supernatant were quantified using a bicinchoninic acid colorimetric assay, in accordance with manufacturer instructions (Pierce Biotechnology, Rockford IL). Samples were assayed in duplicate and were read on a BioTek Synergy H1 hybrid microplate reader (Winooski, VT). Aliquots of tissue lysates sufficient to run duplicates were diluted to 1,000 μg/ml in cell lysis buffer. For serum analyses, samples were simply diluted 1:4 in sample buffer provided by the manufacturer. Immediately before assaying, all samples were centrifuged at 13,000 rcf for 5 min at 4°C to remove any potential debris.

Cytokine and chemokine levels were measured using a Luminex™ 100 suspension array system (Bio-Plex 200, Bio-Rad, Hercules CA) at the University of California, Davis Intellectual and Developmental Disabilities Research Center (IDDRC) core facility. Individual chemokine and cytokine concentrations were calculated using a nine-point standard curve derived from reference cytokines, and the means of animals in each group (*n* = 4–5) were compared. If concentrations fell below the lowest standard, samples were assigned a value the limit of detection/2 for that plate. Because these plates did not include internal reference standards, samples from different plates were not directly compared unless standard curves matched. For visualization and comparisons for animals within a particular age, all cytokine changes were normalized to the geometric mean of the corresponding analyte in FA-exposed female WT animals. As cytokines have different baseline levels, this allowed us to visualize the magnitude of effects of sex, genotype and exposure on all analytes. Absolute cytokines levels were used to compare change as a function of age.

### Statistical Analyses of Multiplex Cytokine Data

Statistical analyses were performed using GraphPad Prism statistical software, version 7.03 (GraphPad Software, La Jolla, CA). Within each time point, each analyte was analyzed separately by three-way ANOVA, using sex, genotype, and exposure as factors. Analyses for the effects of age were analyzed separately, using one-way ANOVA. Our primary goal was to determine the effect(s) of TRAP exposure on cytokine and chemokine levels in the brain and serum. However, we also reported statistically significant main effects of sex or genotype, or an interaction between any of the three ANOVA factors. *Post hoc* analyses were performed using Sidak’s test to correct for multiple tests. Serum cytokine and chemokine concentrations were natural log (ln)-transformed to meet model assumptions of normality and homogeneity of variances. All statistical tests were two-sided and evaluated at a significance level of 0.05.

## Results

### Age Has a Pronounced Tissue-Specific Effect on Hippocampal and Serum Cytokines, Chemokines and Growth Factors

**FIGURE 1:**
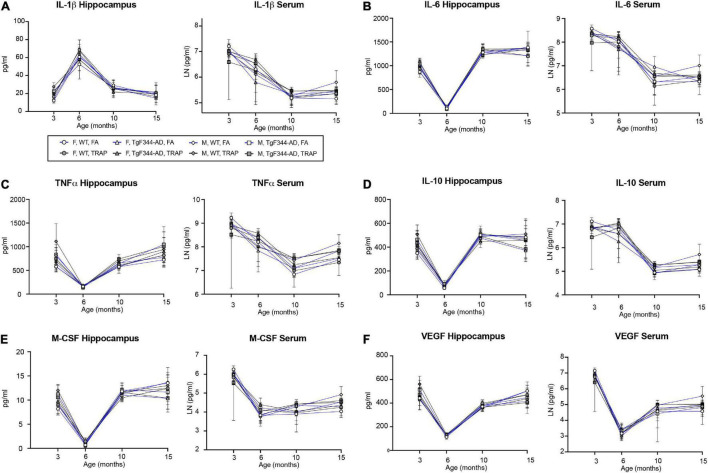
Neither TRAP exposure nor expression of AD risk genes significantly alter age-related changes in cytokines and growth factors in the rat hippocampus and serum. Hippocampal and serum levels of representative cytokines and growth factors in TgF344-AD and WT rats exposed to FA or TRAP beginning at postnatal day 28 and continuing for up to 15 months of age. Representative analytes include **(A)** IL-1β, representative of the inflammasome; **(B)** IL-6 and **(C)** TNFɑ, representative of Th1/pro-inflammatory cytokines; **(D)** IL-10, representative of Th2/anti-inflammatory cytokines; and **(E)** M-CSF and **(F)** VEGF, representative of growth factors. Data points represent the mean ± S.D. (*n* = 4–5 animals per group). Samples were assayed in duplicates; F, females; M, males.

We first evaluated the effect of age on protein expression levels of cytokines in both the serum and the hippocampus of WT and TgF344-AD rats exposed to FA or TRAP (Figure 1). To identify sex differences, mean values in each group were normalized to age-matched female WT animals exposed to FA. The temporal expression profiles varied across cytokines, chemokines and growth factors in the serum and the hippocampus, and for any given cytokine, the expression profile in the serum varied from that of the hippocampus (Figure 1). Analysis of the data using ANOVA identified significant age-dependent differences in expression levels in WT females, results of which are summarized in Supplementary Table 1 (female serum) Supplementary Table 2 (female hippocampus).

In FA WT females, the serum cytokines generally exhibited one of three patterns (Figure 1 and Supplementary Table 1). For IL-1β, IL-18, IL-1ɑ, TNFɑ, IL-6, IL-12 (p70), IL-17, IL-2, IFN_ɣ_, IL-5, IL-10, G-CSF, GM-CSF, and CXCL1, levels were highest at 3 months followed by 6 months and were lowest and approximately equal at 10 and 15 months. The cytokines IL-7, M-CSF, CCL3, CCL20, CCL5, and CCL2 exhibited a pattern in which the highest levels occurred at 3 months, but levels at 6, 10, and 15 were approximately equal. The cytokines IL-4, IL-13, and VEGF were highest at 3 months, followed by 10 and 15 months, with lowest levels observed at 6 months.

In FA WT female, hippocampal cytokines exhibited one of four patterns (Figure 1 and Supplementary Table 2). For IL-1ɑ, IFN_ɣ_, IL-4, IL-5, IL-10, IL-13, G-CSF, M-CSF, CXCL1, CCL3, TNFɑ, and CCL20, approximately equal levels were observed at 3, 10, and 15 months, with lower levels at 6 months. For IL-6, IL-12 (p70), IL-17, IL-7, IL-2, GM-CSF, CCL2, and VEGF, levels were highest at 15 months, followed by 3, 10, and 6 months. TNFɑand CCL5 showed highest levels at 3 months, followed by 15, 10, and 6 months. Finally, IL-1β and IL-18 showed a different profile in which levels were highest at 6 months, followed by approximately equal levels at 3 and 10 months, and lowest levels at 15 months.

### Serum Cytokines Are Not Significantly Altered by Sex, Genotype or Exposure

**FIGURE 2:**
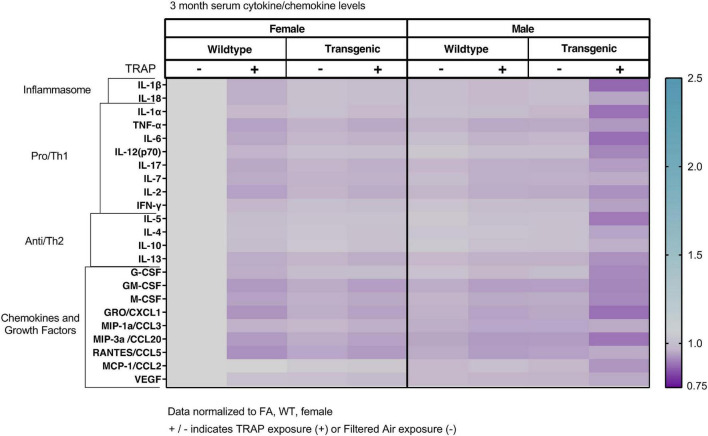
Heat map representation of serum cytokine, chemokine, and growth factor changes in response to TRAP in 3-month old TgF344-AD and WT rats. Serum levels of cytokines and growth factors in TgF344-AD and WT rats exposed to FA or TRAP for 2 months were analyzed using multiplex technology. For each analyte, individual values were normalized to the mean level of that same analyte in sera from female FA WT animals. Purple indicates normalized protein levels < 1.0; turquoise indicates normalized protein levels > 1.0. ± indicates exposure with + identifying TRAP-exposed animals; - indicating FA-exposed animals; *n* = 4–5 animals per group (duplicate samples were analyzed for each animal). Non-normalized cytokine values for female FA WT animals are provided in Supplementary Table 1.

**FIGURE 3:**
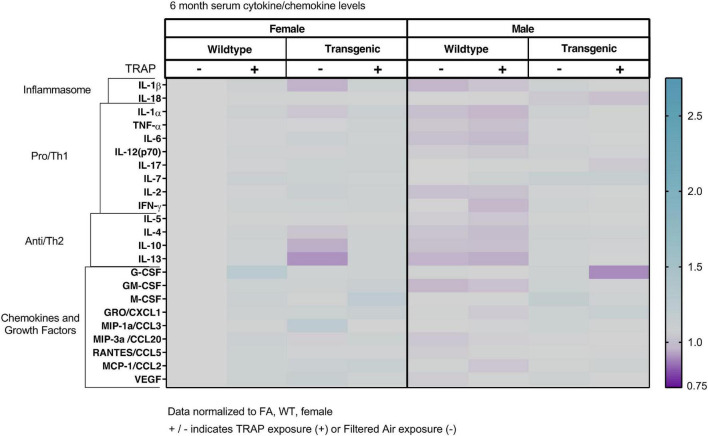
Heat map representation of serum cytokine, chemokine, and growth factor changes in response to TRAP in 6-month old TgF344-AD and WT rats. Serum levels of cytokines and growth factors in TgF344-AD and WT rats exposed to FA or TRAP for 5 months were analyzed using multiplex technology. For each analyte, individual values were normalized to the mean level of that same analyte in sera from female FA WT animals. Purple indicates normalized protein levels < 1.0; turquoise indicates normalized protein levels > 1.0. ± indicates exposure with + identifying TRAP-exposed animals; - indicating FA-exposed animals; *n* = 4–5 animals per group (duplicate samples were analyzed for each animal). Non-normalized cytokine values for female FA WT animals are provided in Supplementary Table 1.

**FIGURE 4:**
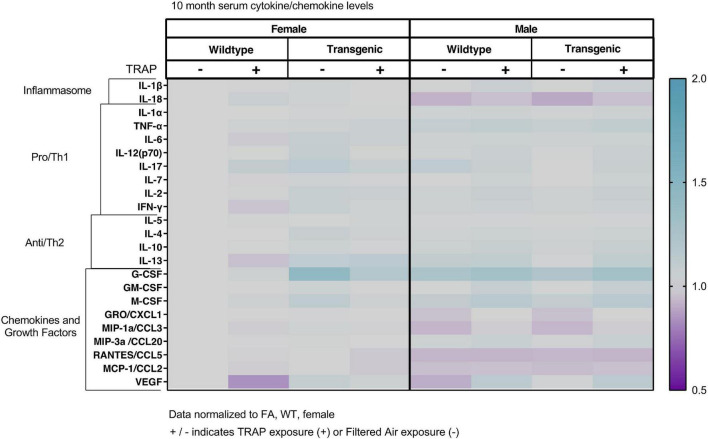
Heat map representation of serum cytokine, chemokine, and growth factor changes in response to TRAP in 10-month old TgF344-AD and WT rats. Serum levels of cytokines and growth factors in TgF344-AD and WT rats exposed to FA or TRAP for 9 months were analyzed using multiplex technology. For each analyte, individual values were normalized to the mean level of that same analyte in sera from female FA WT animals. Purple indicates normalized protein levels < 1.0; turquoise indicates normalized protein levels > 1.0. ± indicates exposure with + identifying TRAP-exposed animals; - indicating FA-exposed animals; *n* = 4–5 animals per group (duplicate samples were analyzed for each animal). Non-normalized cytokine values for female FA WT animals are provided in Supplementary Table 1.

**FIGURE 5:**
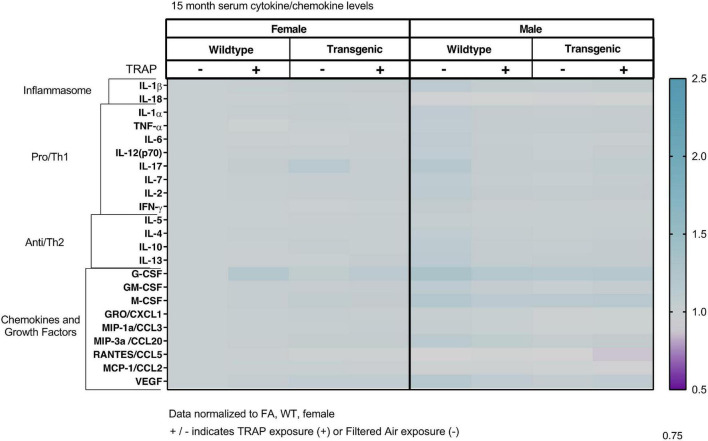
Heat map representation of serum cytokine, chemokine, and growth factor changes in response to TRAP in 15-month old TgF344-AD and WT rats. Serum levels of cytokines and growth factors in TgF344-AD and WT rats exposed to FA or TRAP for 14 months were analyzed using multiplex technology. For each analyte, individual values were normalized to the mean level of that same analyte in sera from female FA WT animals. Purple indicates normalized protein levels < 1.0; turquoise indicates normalized protein levels > 1.0. ± indicates exposure with + identifying TRAP-exposed animals; - indicating FA-exposed animals; *n* = 4–5 animals per group (duplicate samples were analyzed for each animal). Non-normalized cytokine values for female FA WT animals are provided in Supplementary Table 1.

The serum cytokines, chemokines and growth factors were not significantly altered by sex, genotype or exposure in 3 (Figure 2), 6 (Figure 3), 10 (Figure 4), or 15 (Figure 5) month-old animals exposed to FA or TRAP for 2, 5, 9, or 14 months, respectively. Although there appeared to be an overall trend of decreased serum cytokines levels in transgenic males exposed to TRAP at 3 months compared to all other groups (Figure 2), this effect was not significant for any analyte. Cytokine levels were remarkably consistent between groups, and there were no significant effects with any factor (sex, genotype, exposure) at any age (Figures 2–5).

### Hippocampal Cytokines Are Significantly Altered by Sex, Genotype or Exposure in an Age- and Cytokine-Dependent Manner

**FIGURE 6:**
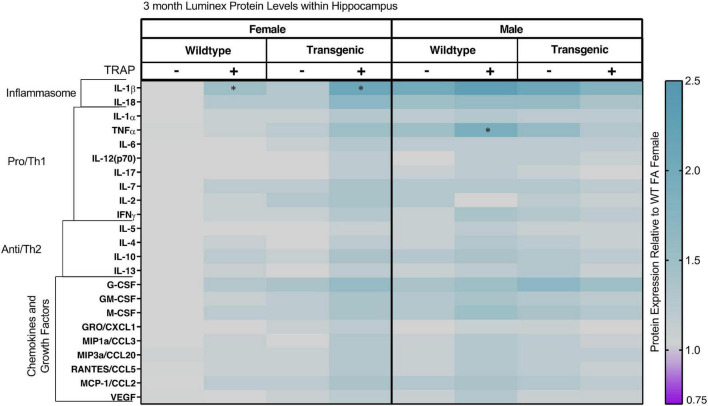
Heat map representation of hippocampal cytokine, chemokine, and growth factor changes in response to TRAP in 3-month old TgF344-AD and WT rats. Hippocampal levels of cytokines and growth factors in TgF344-AD and WT rats exposed to FA or TRAP for 2 months were analyzed using multiplex technology. For each analyte, individual values were normalized to the mean level of that same analyte in hippocampal tissue from female FA WT animals. Purple indicates normalized protein levels < 1.0; turquoise indicates normalized protein levels > 1.0. ± indicates exposure with + identifying TRAP-exposed animals; - indicating FA-exposed animals; *n* = 4–5 animals per group (duplicate samples were analyzed for each animal). *Significantly different from sex-matched WT FA control at *p* < 0.05 as determined by *post hoc* Sidak’s test. Non-normalized cytokine values for female WT animals are provided in Supplementary Table 2.

**FIGURE 7:**
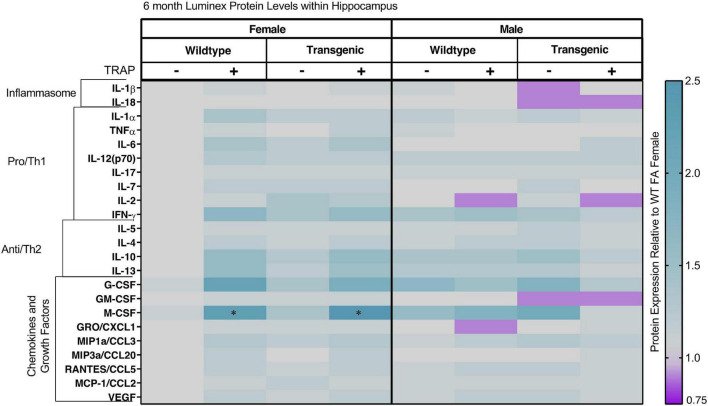
Heat map representation of hippocampal cytokine, chemokine, and growth factor changes in response to TRAP in 6-month old TgF344-AD and WT rats. Hippocampal levels of cytokines and growth factors in TgF344-AD and WT rats exposed to FA or TRAP for 5 months were analyzed using multiplex technology. For each analyte, individual values were normalized to the mean level of that same analyte in hippocampal tissue from female FA WT animals. Purple indicates normalized protein levels < 1.0; turquoise indicates normalized protein levels > 1.0. ± indicates exposure with + identifying TRAP-exposed animals; - indicating FA-exposed animals; *n* = 4–5 animals per group (duplicate samples were analyzed for each animal). *Significantly different from sex-matched WT FA control at *p* < 0.05 as determined by *post hoc* Sidak’s test. Non-normalized cytokine values for female WT animals are provided in Supplementary Table 2.

**FIGURE 8:**
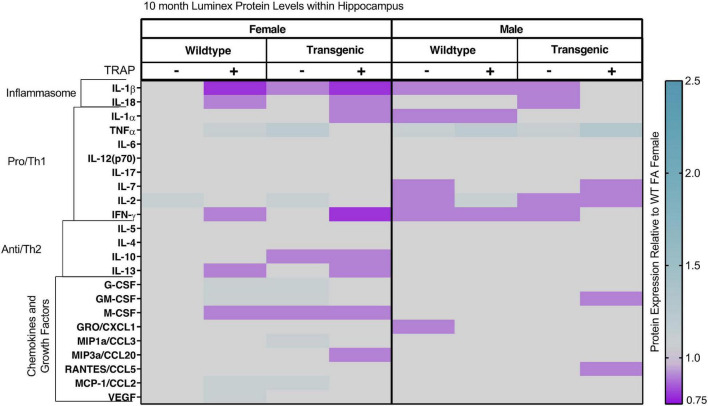
Heat map representation of hippocampal cytokine, chemokine, and growth factor changes in response to TRAP in 10-month old TgF344-AD and WT rats. Hippocampal levels of cytokines and growth factors in TgF344-AD and WT rats exposed to FA or TRAP for 9 months were analyzed using multiplex technology. For each analyte, individual values were normalized to the mean level of that same analyte in hippocampal tissue from female FA WT animals. Purple indicates normalized protein levels < 1.0; turquoise indicates normalized protein levels > 1.0. ± indicates exposure with + identifying TRAP-exposed animals; - indicating FA-exposed animals; *n* = 4–5 animals per group (duplicate samples were analyzed for each animal). Non-normalized cytokine values for female WT animals are provided in Supplementary Table 2.

**FIGURE 9:**
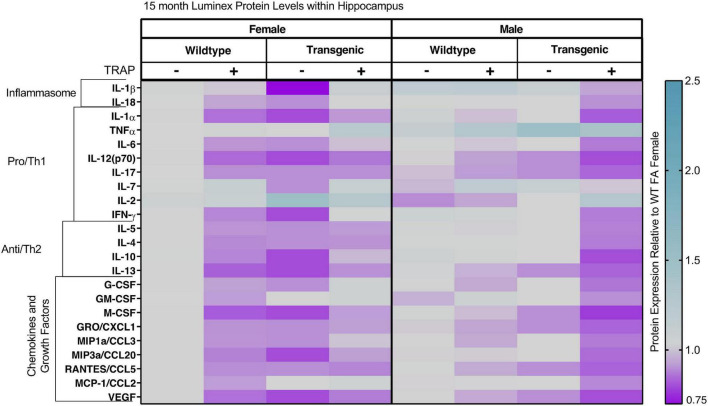
Heat map representation of hippocampal cytokine, chemokine, and growth factor changes in response to TRAP in 15-month old TgF344-AD and WT rats. Hippocampal levels of cytokines and growth factors in TgF344-AD and WT rats exposed to FA or TRAP for 14 months were analyzed using multiplex technology. For each analyte, individual values were normalized to the mean level of that same analyte in hippocampal tissue from female FA WT animals. Purple indicates normalized protein levels < 1.0; turquoise indicates normalized protein levels > 1.0. ± indicates exposure with + identifying TRAP-exposed animals; - indicating FA-exposed animals; *n* = 4–5 animals per group (duplicate samples were analyzed for each animal). Non-normalized cytokine values for female WT animals are provided in Supplementary Table 2.

Heat maps representing group differences in hippocampal cytokines after 2, 5, 9, and 14 months of exposure are shown in Figures 6–9, respectively. In 3-month-old animals exposed to FA or TRAP for 2 months, cytokine levels differed between males and females, and these differences were most pronounced for IL-1b. A 3-way ANOVA (Table 1) revealed a significant main effect of genotype, in which TgF344-AD animals had higher IL-1b than WT animals [*F*(1, 32) = 8.44, *p* = 0.0376]. We also observed a significant main effect of TRAP exposure [*F*(1, 32) = 2.43, *p* = 0.0468] and of sex [*F*(1, 32) = 16.54, *p* < 0.0001], and an interaction between sex and TRAP [*F*(1, 32) = 10.76, *p* = 0.0219]. *Post hoc* analysis showed that TRAP-exposed female TgF344-AD and WT animals had higher levels of IL-1b compared to their FA-exposed controls (1.41-fold in WT animals, *p* = 0.0219; and 1.76-fold in TgF344 animals, *p* = 0.0402, respectively). We also found a significant main effect of sex for TNFɑin 3-month-old animals [*F*(1, 32) = 9.877, *p* = 0.019], and a significant interaction between sex and TRAP exposure [*F*(1, 32) = 12.310, *p* = 0.003]. *Post hoc* analysis showed that male TRAP-exposed animals had higher TNFɑlevels than FA-exposed controls (*p* = 0.0271). No other *post hoc* analyses were significant.

**TABLE 1:** Summary of statistical comparisons for levels of cytokines, chemokines, and growth factors in the hippocampus of 3-month old TgF344-AD and WT rats after 2 months of exposure.

Analyte	Main effect: Sex	Main effect: Genotype	Main effect: TRAP	Interaction	*Post hoc* analysis
IL-1β	*p* < 0.0001	*p* = 0.0376	*p* = 0.0468	Sex*TRAP, *p* = 0.0450	TRAP > FA (F Tg, *p* = 0.0219); TRAP > FA (F WT, *p* = 0.0402)
IL-18	n.s.	n.s.	n.s.	n.s.	—
IL-1ɑ	n.s.	n.s.	n.s.	n.s.	—
TNFɑ	*p* = 0.019	n.s.	n.s.	Sex*TRAP, *p* = 0.003	TRAP > FA (M WT, *p* = 0.0271) F, n.s.
IL-6	n.s.	n.s.	n.s.	n.s.	—
IL-12 (p70)	n.s.	n.s.	n.s.	n.s.	—
IL-17	n.s.	n.s.	n.s.	n.s.	—
IL-7	n.s.	n.s.	n.s.	n.s.	—
IL-2	n.s.	n.s.	n.s.	n.s.	—
IFN_ɣ_	n.s.	n.s.	n.s.	n.s.	—
IL-4	n.s.	n.s.	n.s.	n.s.	—
IL-5	n.s.	n.s.	n.s.	n.s.	—
IL-10	n.s.	n.s.	n.s.	n.s.	—
IL-13	n.s.	n.s.	n.s.	n.s.	—
G-CSF	n.s.	n.s.	n.s.	Sex*Genotype, *p* = 0.0455	n.s.
GM-CSF	n.s.	n.s.	n.s.	n.s.	—
M-CSF	n.s.	n.s.	n.s.	n.s.	—
CXCL1	n.s.	n.s.	n.s.	n.s.	—
CCL3	n.s.	n.s.	n.s.	n.s.	—
CCL20	n.s.	n.s.	n.s.	n.s.	—
CCL5	n.s.	n.s.	n.s.	n.s.	—
CCL2	n.s.	n.s.	n.s.	n.s.	—
VEGF	n.s.	n.s.	n.s.	n.s.	—

*Significant main effects and interactions in three-way ANOVA, and post hoc Sidak’s test, if applicable.*

*“—,” not performed; n.s., not significant; F, Female; M, Male; Tg, TgF344-AD genotype.*

In 6-month-old animals exposed to FA or TRAP (Figure 7 and Table 2), TRAP had an overall effect on the cytokine expression pattern in both TgF344-AD and WT female but not male animals. Statistical analysis indicated there was a significant interaction between sex and TRAP for IFNg (*p* = 0.0492); however, there were no significant *post hoc* comparisons. There was a significant main effect of sex for IL-2 [*F*(1, 32) = 14.056, *p* = 0.002], where males had 0.71-fold lower levels compared to females. In addition, there was an interaction between sex and TRAP for the growth factor G-CSF [*F*(1, 32) = 13.757, *p* = 0.0264], though *post hoc* analyses did not identify significant differences. For the growth factor M-CSF there was a significant interaction between sex, genotype, and TRAP [*F*(1, 32) = 9.256, *p* = 0.0392]. *Post hoc* analyses for M-CSF showed that TRAP-exposed TgF344-AD and WT females had higher levels of M-CSF than their FA-exposed controls (1.53 for TgF344-AD, *p* = 0.0459, and 1.39 for WT, *p* = 0.0398, respectively). There were no additional significant *post hoc* comparisons.

**TABLE 2:** Summary of statistical comparisons for levels of cytokines, chemokines, and growth factors in the hippocampus of 6-month-old TgF344-AD and WT rats exposed to TRAP or FA for 5 months.

Analyte	Main effect: Sex	Main effect: Genotype	Main effect: TRAP	Interaction	*Post hoc* analysis
IL-1β	n.s.	n.s.	n.s.	n.s.	—
IL-18	n.s.	n.s.	n.s.	n.s.	—
IL-1ɑ	n.s.	n.s.	n.s.	n.s.	—
TNFɑ	n.s.	n.s.	n.s.	n.s.	—
IL-6	n.s.	n.s.	n.s.	n.s.	—
IL-12 (p70)	n.s.	n.s.	n.s.	n.s.	—
IL-17	n.s.	n.s.	n.s.	n.s.	—
IL-7	n.s.	n.s.	n.s.	n.s.	—
IL-2	*p* < 0.0001	n.s.	n.s.	n.s.	n.s.
IFN_ɣ_	n.s.	n.s.	*p* = 0.0363	Sex*TRAP, *p* = 0.0492	n.s.
IL-4	n.s.	n.s.	n.s.	n.s.	—
IL-5	n.s.	n.s.	n.s.	n.s.	—
IL-10	n.s.	n.s.	n.s.	n.s.	—
IL-13	n.s.	n.s.	n.s.	n.s.	–
G-CSF	n.s.	n.s.	n.s.	Sex*TRAP, *p* = 0.0264	n.s.
GM-CSF	n.s.	n.s.	n.s.	n.s.	–
M-CSF	n.s.	n.s.	*p* = 0.008	Sex*Genotype* TRAP, *p* = 0.0392	TRAP > FA (F Tg), *p* = 0.0459 TRAP > FA (F WT), *p* = 0.0398
CXCL1	n.s.	n.s.	n.s.	n.s.	—
CCL3	n.s.	n.s.	n.s.	n.s.	—
CCL20	n.s.	n.s.	n.s.	n.s.	—
CCL5	n.s.	n.s.	n.s.	n.s.	—
CCL2	n.s.	n.s.	n.s.	n.s.	—
VEGF	n.s.	n.s.	n.s.	n.s.	—

*Significant main effects and interactions in three-way ANOVA, and post hoc Sidak’s test, if applicable.*

*“—,” not performed; n.s., not significant; F, Female; M, Male; Tg, TgF344-AD genotype.*

In 10- and 15-month-old animals, sex, genotype and exposure had minimal effects on levels of cytokines, chemokines and growth factors (Figures 8, 9 and Tables 3, 4). In 10-month-old animals, TRAP exposure for 9 months increased levels of IL-1b compared to FA-exposed animals [*F*(1, 32) = 2.11, *p* = 0.0491]. However, there were no significant *post hoc* analyses and changes were restricted to a main effect. Similarly, we did not observe any clear effects of genotype or TRAP exposure on most analytes (Figure 9). The only significant effects was observed for M-CSF, where there was a significant interaction between TRAP and genotype [*F*(1, 32) = 13.482, *p* = 0.0233]. However, there were no significant *post hoc* analyses.

**TABLE 3:** Summary of statistical comparisons for levels of cytokines, chemokines, and growth factors in the hippocampus of 10-month-old TgF344-AD and WT rats exposed to TRAP or FA for 9 months.

Analyte	Main effect: Sex	Main effect: Genotype	Main effect: TRAP	Interaction	*Post hoc* analysis
IL-1β	n.s.	n.s.	*p* = 0.0491	n.s.	n.s.
IL-18	n.s.	n.s.	n.s.	n.s.	—
IL-1ɑ	n.s.	n.s.	n.s.	n.s.	—
TNFɑ	n.s.	n.s.	n.s.	n.s.	—
IL-6	n.s.	n.s.	n.s.	n.s.	—
IL-12 (p70)	n.s.	n.s.	n.s.	n.s.	—
IL-17	n.s.	n.s.	n.s.	n.s.	—
IL-7	n.s.	n.s.	n.s.	n.s.	—
IL-2	n.s.	n.s.	n.s.	n.s.	—
IFN_ɣ_	n.s.	n.s.	n.s.	n.s.	—
IL-4	n.s.	n.s.	n.s.	n.s.	—
IL-5	n.s.	n.s.	n.s.	n.s.	—
IL-10	n.s.	n.s.	n.s.	n.s.	—
IL-13	n.s.	n.s.	n.s.	n.s.	—
G-CSF	n.s.	n.s.	n.s.	n.s.	—
GM-CSF	n.s.	n.s.	n.s.	n.s.	—
M-CSF	n.s.	n.s.	n.s.	n.s.	—
CXCL1	n.s.	n.s.	n.s.	n.s.	—
CCL3	n.s.	n.s.	n.s.	n.s.	—
CCL20	n.s.	n.s.	n.s.	n.s.	—
CCL5	n.s.	n.s.	n.s.	n.s.	—
CCL2	n.s.	n.s.	n.s.	n.s.	—
VEGF	n.s.	n.s.	n.s.	n.s.	—

*Significant main effects and interactions in three-way ANOVA, and post hoc Sidak’s test, if applicable.*

*“—,” not performed; n.s., not significant; F, Female; M, Male; Tg, TgF344-AD genotype.*

**TABLE 4:** Summary of statistical comparisons for levels of cytokines, chemokines, and growth factors in the hippocampus of 15-month-old TgF344-AD and WT rats exposed to TRAP or FA for 14 months.

Analyte	Main effect: Sex	Main effect: Genotype	Main effect: TRAP	Interaction	*Post hoc* analysis
IL-1β	n.s.	n.s.	n.s.	n.s.	—
IL-18	n.s.	n.s.	n.s.	n.s.	—
IL-1ɑ	n.s.	n.s.	n.s.	n.s.	—
TNFɑ	n.s.	n.s.	n.s.	n.s.	—
IL-6	n.s.	n.s.	n.s.	n.s.	—
IL-12 (p70)	n.s.	n.s.	n.s.	n.s.	—
IL-17	n.s.	n.s.	n.s.	n.s.	—
IL-7	n.s.	n.s.	n.s.	n.s.	—
IL-2	n.s.	n.s.	n.s.	n.s.	—
IFN_ɣ_	n.s.	n.s.	n.s.	n.s.	—
IL-4	n.s.	n.s.	n.s.	n.s.	—
IL-5	n.s.	n.s.	n.s.	n.s.	—
IL-10	n.s.	n.s.	n.s.	n.s.	—
IL-13	n.s.	n.s.	n.s.	n.s.	–
G-CSF	n.s.	n.s.	n.s.	n.s.	–
GM-CSF	n.s.	n.s.	n.s.	n.s.	–
M-CSF	n.s.	n.s.	n.s.	TRAP*Genotype, *p* = 0.0233	n.s.
CXCL1	n.s.	n.s.	n.s.	n.s.	—
CCL3	n.s.	n.s.	n.s.	n.s.	—
CCL20	n.s.	n.s.	n.s.	n.s.	—
CCL5	n.s.	n.s.	n.s.	n.s.	—
CCL2	n.s.	n.s.	n.s.	n.s.	—
VEGF	n.s.	n.s.	n.s.	n.s.	—

*Significant main effects and interactions in three-way ANOVA, and post hoc Sidak’s test, if applicable.*

*“—,“ not performed; n.s., not significant; F, Female; M, Male; Tg, TgF344-AD genotype.*

## Discussion

In this study, we assayed tissue previously collected from TgF344-AD and WT animals exposed to TRAP or FA (Patten et al., 2021) to determine the impacts of age, TRAP exposure, and AD genetic susceptibility on cytokine and chemokine profiles in the serum and hippocampus. We also asked whether these factors had similar effects on cytokine and chemokine expression in the serum and hippocampus. The primary findings were: (1) Both serum and hippocampal cytokines were strongly affected by age, but specific temporal expression patterns varied by analyte, and for each individual analyte, differed between serum and hippocampus. (2) TRAP and genotype had no significant effects on the expression of cytokines in the serum at any of the ages we examined. (3) For hippocampal cytokines, TRAP generally had a stronger effect than sex or genotype. (4) The effects of TRAP on hippocampal cytokines were predominantly seen at younger ages (3 and 6 months).

Age was the most significant factor influencing cytokine/chemokine/growth factor levels in both the hippocampus and serum. These age-specific differences highlight the complexity of cytokine responses in TgF344-AD and WT rats over time and emphasize the need to exercise caution when interpreting the significance of cytokine changes for a single time-point in preclinical AD studies. Our observation that temporal expression patterns of serum and hippocampal cytokines did not mirror one another suggests that in this model, serum does not correspond with changes in CNS cytokine levels. This observation is not without precedent: peripheral immune challenges have been shown to stimulate pronounced inflammatory responses, including altered cytokine levels, that vary significantly between the serum and brain in aged mice (Barrientos et al., 2009) and juvenile rats (Bruce et al., 2019). These data suggest that serum cytokine profiles will not be a useful proxy or biomarker of brain cytokine levels.

Immune dysregulation is a well-characterized feature of aging (Rea et al., 2018), and historically has been associated with increased levels of pro-inflammatory cytokines, and decreased levels of anti-inflammatory cytokines (Michaud et al., 2013b; Charlton et al., 2018). Thus, it was somewhat unexpected that we did not observe significantly increased levels of pro-inflammatory cytokines with increasing age in either the hippocampus or the serum of TgF344-AD or WT rats. This is particularly surprising in the hippocampus, as many rodent studies have reported increased levels of pro-inflammatory cytokines in aged animals compared to young animals (Lynch, 2010; Barrientos et al., 2015). In humans, there are few postmortem studies comparing cytokine levels in the brain of healthy aged vs. young individuals, but cytokine measurements in CSF follow a trend similar to what is observed in rodent brain with aged individuals typically exhibiting higher and lower levels of pro- and anti-inflammatory cytokines, respectively, relative to younger controls (Hu et al., 2019). Human data of serum cytokine levels are similar, although several studies have reported no change in serum cytokines with age (Beharka et al., 2001), or have reported increases only in individuals > 65 years (Wyczalkowska-Tomasik et al., 2016). In our study, it is possible that these animals were not old enough to observe a significant increase in serum or hippocampal cytokine levels. Alternatively, it is possible that the immune changes in the brain are not mediated primarily by cytokines in rat models of aging.

AD has also been associated with increased levels of pro-inflammatory cytokines such as IL-1β, IL-6, and TNFɑ(Cacquevel et al., 2004). Elevated levels of these cytokines have been detected in postmortem brain from AD patients proximal to amyloid-β (Aβ) plaques (Zheng et al., 2016). We previously identified Aβ plaques and oligomers in TgF344-AD rats that increased with age, as well as significantly increased astrocyte reactivity and microglial activation in the hippocampus of TgF344-AD rats relative to age- and sex-matched WT littermates (Patten et al., 2021). Aβ plaques and oligomers are known to activate microglia to release cytokines, thus, it was surprising that we did not observe differences in hippocampal cytokines between WT and TgF344-AD animals at later ages, when TgF344-AD rats have numerous plaques. While cytokine and chemokine levels in the serum and hippocampus have not been previously evaluated in the TgF344-AD rat model, a recent study of cytokine expression in the brain stem of the TgF344-AD rat model is consistent with our findings. The authors reported no differences in brain stem levels of the cytokines IFN-γ, IL-1β, IL-4, IL-5, IL-10, IL-13, KC, and TNFɑbetween TgF344-AD and WT rats aged 8–11 months (Lucking et al., 2019). In contrast, the APP/PS1 mouse, which shares the same genetic mutations as the TgF344-AD rat, exhibited modest increases in TNFɑ, IL-1ɑ, and IL-1Rɑat 9 months compared to WT mice (Babcock et al., 2015). This may reflect species differences given emerging evidence that neuroinflammatory responses in the rat and mouse diverge significantly (Leist and Hartung, 2013; Seok et al., 2013; Zhang et al., 2016; Du et al., 2017; Kodamullil et al., 2017), with the rat more closely resembling the human neuroinflammatory response (Du et al., 2017).

Our findings are consistent with recent human studies that find inconsistent changes in cytokine levels in AD patients. A longitudinal study of patients with mild cognitive impairment (MCI) showed that serum levels of IFNγ, IL−1β, IL−2, IL−4, IL−6, and IL−10 decreased over time, and these decreases were highly correlated with decreased cognition (Thomas et al., 2020), and a study of post-mortem samples reported that relative to non-demented controls, AD brains and serum had decreased levels of IL-1b, IL-6, IL-7, IL-8, and IL-16 (Chen et al., 2016). Interestingly, a longitudinal study of patients with mild cognitive impairment (MCI) who progressively developed AD found that PET scans of neuroinflammation identified two peaks of neuroinflammation: one peak early in disease progression, and a second at a late stage (Fan et al., 2017). Specifically, in comparison to normal controls, patients with MCI initially had increased levels of neuroinflammation; however, this was followed by a period of decreased neuroinflammation that persisted for 14 months. Long-term, patients who developed AD showed a second increase in neuroinflammation (Fan et al., 2017). Although cytokines were not measured in this study, it is possible that a similar alternating pattern of cytokine expression may occur. It will be important in future work to determine whether hippocampal cytokines, particularly pro-inflammatory cytokines, increase significantly in animals older than 15 months and whether this is enhanced in animals with advanced AD pathology.

TRAP exposure had no effect on serum cytokine levels, but did significantly increase levels of a subset of cytokines in the hippocampus of 3- and 6-month-old animals. Specifically, at 3 months TRAP significantly increased levels of IL-1β in the hippocampus of female TgF344-AD and WT rats. IL-1β is a potent activator of astrocytes and microglia in the CNS (Mrak and Griffin, 2001; Veryard et al., 2012; Scarabino et al., 2020). While IL-1β is expressed at low levels in the healthy CNS, it can be rapidly induced in response to injury (Liu and Quan, 2018). In AD, the IL-1 family has been of interest due to its close association with neuroinflammation (Shaftel et al., 2008; Italiani et al., 2018; Liu and Quan, 2018), the presence of genetic mutations in IL-1 that link to AD (Licastro et al., 2004; Rainero et al., 2004), and the presence of IL-1 cytokines in AD brains (Swanson et al., 2018). More recently, work has implicated IL-1β very early in AD pathology through its role in the inflammasome (Heneka, 2017). Inflammasomes are innate immune system sensors that are activated in response to damage or danger signals (Heneka, 2017; Venegas and Heneka, 2017), and one hallmark of this is IL-1β and IL-18 release (van de Veerdonk et al., 2011). In AD, inflammasome activation has been shown to seed Aβ plaques and contribute to Aβ plaque pathology (Heneka, 2017; Venegas et al., 2017; Tejera et al., 2019). We previously showed that TRAP promoted Aβ deposition in the hippocampus of 3-month old TgF344-AD females, in contrast to 3 month-old TgF344-AD rats exposed to FA or age-matched TgF344-AD males exposed to either TRAP or FA that showed no Aβ deposition (Patten et al., 2021). The observation of increased IL-1β in the brains of these same animals suggests that TRAP promotes AD phenotypes *via* activation of the inflammasome, a hypothesis that will need to be confirmed in future work.

Although we observed that TRAP increased hippocampal levels of IL-1β in females and TNFβ in males at 3 months, by 6 months these effects had disappeared, and we instead found that TRAP significantly increased hippocampal levels of M-CSF in WT and TgF344-AD females. M-CSF is a hematopoietic growth factor that increases proliferation and phagocytosis of microglia in the CNS (Mitrasinovic et al., 2003; Pons et al., 2020). Its expression in the brain has also been shown to promote recruitment of peripheral monocytes and macrophages into the CNS (Mitrasinovic et al., 2005; Boissonneault and Rivest, 2009; Lampron et al., 2013; Daria et al., 2017; Pons et al., 2020). As IL-1β was elevated in the hippocampus of female TRAP-exposed rats at 3 months of age, coincident with increased AD pathology (Patten et al., 2021), one possibility is that an increase in M-CSF indicates a response of the immune system to AD pathology and inflammation (Elmore et al., 2014; Dagher et al., 2015; Sasaki, 2016; Unger et al., 2018). We did not observe this effect in TRAP-exposed male animals of either genotype, highlighting sex-specific responses to TRAP independent of genotype. Additionally, sex-specific differences included a significant effect of sex on hippocampal IL-2 levels at 6 months, with males exhibiting lower levels. IL-2 is typically produced by T cells, but in the brain can also be expressed by hippocampal neurons (Meola et al., 2013). Although its role in the context of AD is not clear, decreased levels of IL-2 in the hippocampus have been associated with exacerbated AD pathology in mice (Alves et al., 2017), and treatment of AD mice with exogenous IL-2 significantly reduced AD pathology *via* increased regulatory T cell activity (Dansokho et al., 2016; Alves et al., 2017). Although both species and sex used in these studies differed from our own, collectively our data support further investigation into the role of IL-2 in exacerbating AD pathology. At 10 and 15 months, we observed minimal effects of TRAP on hippocampal cytokines. At 10 months, IL-1β in the hippocampus was decreased in response to TRAP; at 15 months, we observed a significant interaction between TRAP and genotype, but *post hoc* analyses did not identify any significant differences between groups. Overall, our cytokine data are supported by our previous studies showing that TRAP activated microglia most robustly in 3- and 6-month-old animals (Patten et al., 2021). Although we did not determine the source of IL-1β in this study, IBA1 + microglia are thought to be the most significant producers of IL-1β in the CNS (Griffin and Barger, 2010; Italiani et al., 2018), and are a major source of many other cytokines.

In summary, this study revealed a strong effect of age on both hippocampal and serum cytokines, but indicated these effects were non-linear. However, a caveat to this conclusion is that multiple factors can influence cytokines, including differences in season. Because of the long exposure time (14 months), animals at different ages were exposed to different seasonal effects. Seasonal effects are known to affect cytokine levels, and are linked to changes in diseases such as multiple sclerosis (Ter Horst et al., 2016). In addition, a previous study of cardiovascular disease patients found that cytokine levels are much lower in the spring (Spath et al., 2017). In this study, animals aged 6 months were euthanized in the spring, and in our analyses, we found that many cytokines showed strikingly different values at 6 months, compared with other ages, although it is noted that cytokines were not universally decreased at 6 months, and indeed, hippocampal IL-1β levels were significantly increased at this age relative to earlier and later ages. Therefore, while it is tempting to ascribe purely age-dependent changes to serum and hippocampal cytokines, future work is necessary to determine whether these results were confounded by season. Second, we showed that serum cytokine patterns did not mirror hippocampal cytokines, suggesting that serum cytokines may have limited prognostic value in assessing CNS inflammation. While CSF cytokine profiles are often highly correlated with levels in brain tissue (Mitrasinovic et al., 2003; Choi et al., 2008; Olson and Humpel, 2010), obtaining CSF requires a lumbar puncture and therefore is not routinely evaluated in AD patients, and it is difficult to obtain sufficient quantities for analysis from rodents. Thus, CSF cytokine levels would be challenging as a routine biomarker. Third, within each age group, TRAP exposure had a stronger effect on cytokines than either genotype or sex. The most significant TRAP-induced cytokine changes occurred in 3- and 6-month-old animals, suggesting the possibility of early inflammatory changes as a key factor mediating TRAP effects on AD.

## Data Availability Statement

The raw data supporting the conclusions of this article will be made available by the authors, without undue reservation.

## Ethics Statement

The animal study was reviewed and approved by the University of California, Davis Institutional Animal Care and Use Committee.

## Author Contributions

PL, KB, and AW obtained funding to support the work and supervised all aspects of the study. KB, CW, and AW constructed the tunnel vivarium and exposure chambers. AV maintained the rat colony. AV, KP, CW, and KB maintained the animals at the tunnel facility. KP and PL designed the experiments. AV and KP collected tissues for analysis. KP conducted the cytokine analyses, immunohistochemistry, image acquisition and image analysis, and drafted the initial manuscript, including the figures. DH conducted the statistical analyses of the data. FG and PL made significant edits to the early versions of the manuscript. All authors reviewed and made final edits to the manuscript prior to submission.

## Conflict of Interest

The authors declare that the research was conducted in the absence of any commercial or financial relationships that could be construed as a potential conflict of interest.

## Publisher’s Note

All claims expressed in this article are solely those of the authors and do not necessarily represent those of their affiliated organizations, or those of the publisher, the editors and the reviewers. Any product that may be evaluated in this article, or claim that may be made by its manufacturer, is not guaranteed or endorsed by the publisher.

## Abbreviations

Aβ: amyloid-β
CSF: cerebrospinal fluid
FA: filtered air
G-CSF: granulocyte colony-stimulating factor
GM-CSF: granulocyte-macrophage colony-stimulating factor
GROɑ/CXCL-1/KC: growth-regulated protein alpha/C-X-C motif ligand 1/keratinocyte chemoattractant
IL: interleukin
IFN: interferon
M-CSF: macrophage colony-stimulating factor
MCP-1/CCL2: monocyte chemoattractant protein 1/C-C motif ligand 2
MIP-1ɑ: macrophage inflammatory protein-1 alpha
PBS: phosphate buffered saline
RANTES: regulated upon activation, normal T cells, expressed and secreted
TGF-β: transforming-growth factor beta
TNF: tumor necrosis factor
TRAP: traffic-related air pollution
VEGF: vascular endothelial growth factor
WT: wildtype.
