# Supplementary material for: Hippocampal but Not Serum Cytokine Levels Are Altered by Traffic-Related Air Pollution in TgF344-AD and Wildtype Fischer 344 Rats in a Sex- and Age-Dependent Manner
Source: Front Cell Neurosci. 2022 Apr 22;16:861733. doi: 10.3389/fncel.2022.861733 (PMC9072828; doi:10.3389/fncel.2022.861733)
Supplement: Supplementary file 1 [file Data_Sheet_1.pdf]

Supplementary Table S1. Summary of statistical comparisons for levels of cytokines, chemokines, and growth factors in the serum of WT rats exposed to FA for 2, 5, 9, or 14 months.

Analyte	Sex	3 mo avg. pg/ml +/- s.d.	6 mo avg. pg/ml +/- s.d.	10 mo avg. pg/ml +/- s.d.	15 mo avg. pg/ml +/- s.d.	Significant differences by age* ("pattern")
IL-1β	F	1355.93 ± 395.41	550.28 ± 226.36	178.96 ± 55.96	174.09 ± 29.88	3 vs 6, 3 vs 10, 3 vs 15, 6 vs 10, 6 vs 15
	M	1090.91 ± 203.56	508.88 ± 310.46	193.19 ± 57.82	330.14 ± 191.15	
IL-18	F	10295.03 ± 3233.85	7811.23 ± 924.39	5238.85 ± 2551.32	6142.02 ± 1004.72	3 vs 6, 3 vs 10, 3 vs 15, 6 vs 10, 6 vs 15
	M	7937.63 ± 677.42	8941.67 ± 536.83	2479.80 ± 828.62	3698.45 ± 2081.84	
IL-1α	F	1437.40 ± 293.57	950.92 ± 259.45	624.69 ± 155.29	632.75 ± 80.44	3 vs 6, 3 vs 10, 3 vs 15, 6 vs 10, 6 vs 15
	M	1089.52 ± 147.43	850.82 ± 352.11	760.86 ± 227.75	1274.51 ± 724.29	
TNFα	F	10169.00 ± 2291.14	3662.24 ± 1278.52	929.61 ± 384.92	1723.97 ± 144.36	3 vs 6, 3 vs 10, 3 vs 15, 6 vs 10, 6 vs 15, 10 vs 15
	M	7927.83 ± 1255.39	3072.18 ± 1774.42	1694.51 ± 553.15	3471.77 ± 1518.86	
IL-6	F	5283.71 ± 870.58	3051.81 ± 1033.77	557.54 ± 230.80	561.23 ± 62.06	3 vs 6, 3 vs 10, 3 vs 15, 6 vs 10, 6 vs 15
	M	4507.59 ± 371.96	2499.07 ± 1478.59	700.98 ± 165.65	1107.77 ± 621.20	
IL-12 (p70)	F	5891.10 ± 1192.12	3325.21 ± 1354.06	1007.71 ± 701.46	834.35 ± 148.62	3 vs 6, 3 vs 10, 3 vs 15, 6 vs 10, 6 vs 15
	M	4330.49 ± 452.08	3091.68 ± 2054.66	1067.44 ± 310.12	1648.06 ± 1010.57	
IL-17	F	328.95 ± 61.97	42.37 ± 18.45	21.31 ± 10.90	22.79 ± 1.37	3 vs 6, 3 vs 10, 3 vs 15, 6 vs 10
	M	276.35 ± 27.42	40.34 ± 4.31	35.42 ± 29.40	44.07 ± 27.21	
IL-7	F	1600.59 ± 327.13	124.43 ± 81.44	143.63 ± 43.15	141.47 ± 17.88	3 vs 6, 3 vs 10, 3 vs 15
	M	1207.77 ± 145.25	118.61± 59.93	146.23 ± 41.70	253.63 ± 151.94	
IL-2	F	14712.12 ± 2644.81	10008.28 ± 2992.49	1767.72 ± 902.65	1594.38 ± 231.00	3 vs 6, 3 vs 10, 3 vs 15, 6 vs 10, 6 vs 15
	M	12071.05 ± 1364.23	7747.17 ± 5167.75	2510.53 ± 972.51	3959.03 ± 2533.52	
IFNɣ	F	5788.55 ± 805.39	1369.53 ± 562.63	642.99 ± 225.59	776.46 ± 41.30	3 vs 6, 3 vs 10, 3 vs 15, 6 vs 10, 6 vs 15
	M	5034.42 ± 338.07	1132.17 ± 779.75	776.18 ± 163.51	1326.06 ± 755.76	
IL-4	F	1167.92 ± 292.79	392.25 ± 86.70	408.51 ± 135.31	409.85 ± 36.30	3 vs 6, 3 vs 10, 3 vs 15, 6 vs 10, 6 vs 15
	M	1029.65 ± 77.80	345.01 ± 232.22	447.27 ± 114.28	757.60 ± 471.20	
IL-5	F	1895.74 ± 201.93	1336.92 ± 302.64	636.27 ± 82.59	633.34 ± 27.00	3 vs 6, 3 vs 10, 3 vs 15, 6 vs 10, 6 vs 15
	M	1640.37 ± 88.35	1230.75 ± 365.51	743.42 ± 178.77	879.79 ± 284.89	
IL-10	F	1225.94 ±209.68	858.33 ± 298.19	140.12 ± 36.16	159.87 ± 13.98	3 vs 6, 3 vs 10, 3 vs 15, 6 vs 10, 6 vs 15
	M	978.81 ±111.81	721.10 ± 457.37	177.65 ± 45.06	301.71 ± 164.46	
IL-13	F	3207.63 ± 590.10	1204.43 ± 957.11	489.34 ± 274.20	500.21 ± 59.19	3 vs 6, 3 vs 10, 3 vs 15, 6 vs 10, 6 vs 15
	M	2680.27 ± 345.25	1089.68 ± 791.18	818.77 ± 247.50	1172.64 ± 586.68	
G-CSF	F	177.39 ± 31.10	67.55 ± 28.76	11.88 ± 8.15	13.63 ± 0.53	3 vs 6, 3 vs 10, 3 vs 15, 6 vs 10, 6 vs 15
	M	137.78 ± 13.65	64.36 ± 39.65	18.58 ± 5.38	34.62 ± 19.99	
GM-CSF	F	1060.72 ± 205.25	191.98 ± 82.59	98.71 ± 38.56	98.46 ± 16.48	3 vs 6, 3 vs 10, 3 vs 15, 6 vs 10, 6 vs 15
	M	809.29 ± 96.78	163.56 ± 117.37	102.57 ± 33.44	181.99 ± 118.81	
M-CSF	F	521.55 ± 104.03	48.97 ± 18.76	48.97 ± 20.50	56.44 ± 11.66	3 vs 6, 3 vs 10, 3 vs 15
	M	402.75 ± 51.11	42.39 ± 8.89	72.03 ± 24.01	136.84 ± 72.63	
CXCL1	F	472.41 ± 59.04	182.76 ± 76.56	222.89 ± 21.49	215.92 ± 21.65	3 vs 6, 3 vs 10, 3 vs 15, 6 vs 10
	M	358.66 ± 34.45	267.68 ± 111.93	181.11 ± 45.44	263.34 ± 113.79	
CCL3	F	247.07 ± 46.23	27.68 ± 7.88	35.60 ± 5.37	40.15 ± 2.53	3 vs 6, 3 vs 10, 3 vs 15, 6 vs 15
	M	180.84 ± 18.64	26.36 ± 4.03	28.51 ± 7.98	48.97 ± 18.42	
CCL20	F	199.26 ± 38.14	33.21 ± 54.34	23.48 ± 1.73	24.77 ± 2.36	3 vs 6, 3 vs 10, 3 vs 15
	M	152.91 ± 17.73	21.38 ± 17.56	25.96 ± 5.11	40.27 ± 20.05	

CCL5	F	1318.80 ± 71.86	600.98 ± 188.12	802.32 ± 135.57	821.79 ± 143.77	3 vs 6, 3 vs 10, 3 vs 15, 6 vs 10, 6 vs 15
	M	1060.87 ± 97.64	547.27 ± 94.14	501.45 ± 119.93	429.29 ± 74.94	
CCL2	F	2468.57 ± 322.65	788.23 ± 11.43	956.60 ± 124.75	975.76 ± 48.25	3 vs 6, 3 vs 10, 3 vs 15, 6 vs 10, 6 vs 15
	M	1930.92 ± 121.83	723.44 ± 179.98	682.56 ± 139.89	814.33 ± 251.99	
VEGF	F	1267.15 ± 244.74	23.45 ± 10.01	96.15 ± 62.24	97.65 ± 20.39	3 vs 6, 3 vs 10, 3 vs 15, 6 vs 10, 6 vs 15
	M	977.67 ± 131.82	20.25 ± 3.12	142.79 ± 19.33	254.15 ± 212.31	

*Significant differences and direction determined by one-way ANOVA.

Supplementary Table S2. Summary of statistical comparisons for levels of cytokines, chemokines, and growth factors in the hippocampus of WT rats exposed to FA for 2, 5, 9, or 14 months.

Analyte	Sex	3 mo avg. pg/ml +/- s.d.	6 mo avg. pg/ml +/- s.d.	10 mo avg. pg/ml +/- s.d.	15 mo avg. pg/ml +/- s.d.	Significant age comparisons ($p<0.05$)*
IL-1β	F	20.13 ± 8.19	60.60 ± 4.53	29.14 ± 5.69	18.63 ± 4.04	3 vs 6, 3 vs 10, 6 vs 10, 6 vs 15, 10 vs 15
	M	29.61 ± 2.09	66.01 ± 8.01	25.73 ± 3.17	20.13 ± 8.19	
IL-18	F	224.75 ± 11.94	261.28 ± 20.68	166.93 ± 12.23	200.12 ± 6.32	3 vs 10, 3 vs 15, 6 vs 10, 6 vs 15, 10 vs 15
	M	237.13 ± 30.63	267.68 ± 21.71	160.22 ± 10.49	201.67 ± 20.83	
IL-1α	F	56.25 ± 2.39	7.81 ± 1.89	54.89 ± 4.05	55.18 ± 6.13	3 vs. 6, 6 vs 10, 6 vs 15
	M	67.50 ± 11.42	9.09 ± 1.52	49.90 ± 3.06	58.71 ± 4.56	
TNFα	F	592.28 ± 193.50	157.49 ±24.48	576.29 ± 136.28	719.99 ± 54.88	3 vs 6, 6 vs 10, 6 vs 15, 10 vs 15
	M	1198.42 ± 237.82	174.37 ± 26.88	593.41 ± 33.73	796.47 ± 236.44	
IL-6	F	1073.83 ± 66.88	100.49 ± 16.26	1279.26 ± 88.38	1383.94 ± 94.58	3 vs 6, 3 vs 10, 3 vs 15, 6 vs 10, 6 vs 15
	M	1268.60 ± 145.58	113.04 ± 18.48	1233.79 ± 53.45	1388.77 ± 83.33	
IL-12 (p70)	F	501.9 ± 42.71	82.02 ± 14.37	367.42 ±17.23	514.51 ± 50.84	3 vs 6, 3 vs 10, 6 vs 10, 6 vs 15, 10 vs 15
	M	508.32 ± 106.02	96.39 ± 11.43	365.56 ± 25.15	510.01 ± 41.51	
IL-17	F	84.68 ± 6.17	29.19 ± 2.33	75.20 ± 3.14	97.57 ± 10.08	3 vs 6, 6 vs 10, 6 vs 15, 10 vs 15
	M	94.15 ± 13.32	31.34 ± 1.90	75.18 ± 2.64	94.55 ± 6.22	
IL-7	F	211.43 ± 6.17	45.11 ± 6.19	187.11 ± 37.77	265.32 ± 47.56	3 vs 6, 3 vs 15, 6 vs 10, 6 vs 15
	M	271.86 ± 20.57	42.68 ± 4.30	166.65 ± 7.73	241.57 ± 60.13	
IL-2	F	770.46 ± 63.84	457.36 ± 81.59	2038.35 ±875.99	1313.89 ± 545.42	3 vs 6, 3 vs 10, 6 vs 10, 6 vs 15, 10 vs 15
	M	1001.60 ± 188.17	460.73 ± 139.58	1607.26 ± 546.04	1174.52 ± 116.45	
IFNɣ	F	6777.24 ± 362.62	674.45 ± 106.88	6400.44 ± 599.31	6278.50 ± 724.36	3 vs 6, 6 vs 10, 6 vs 15
	M	7444.96 ± 1156.33	943.63 ± 247.89	5685.23 ± 233.06	6668.43 ± 990.36	
IL-4	F	51.57 ± 3.27	12.30 ± 1.03	50.20 ± 4.69	54.62 ± 5.22	3 vs 6, 6 vs 10, 6 vs 15
	M	56.73 ± 8.64	13.79 ± 0.93	48.08 ± 1.77	55.19 ± 5.35	
IL-5	F	150.54 ± 6.14	40.69 ± 3.47	157.53 ± 7.43	153.80 ± 8.04	3 vs 6, 6 vs 10, 6 vs 15
	M	165.59 ± 7.80	43.75 ± 4.05	161.42 ± 6.75	147.92 ± 9.77	
IL-10	F	529.26 ± 59.91	56.39 ± 12.55	500.97 ± 34.95	478.16 ± 71.93	3 vs 6, 3 vs 15, 6 vs 10, 6 vs 15
	M	688.01 ± 103.86	99.41 ± 20.71	488.29 ± 42.81	498.36 ± 117.16	
IL-13	F	115.76 ± 5.72	10.02 ± 2.50	96.66 ± 6.47	108.74 ± 10.28	3 vs 6, 3 vs 10, 6 vs 10, 6 vs 15
	M	127.34 ± 15.63	12.36 ± 5.26	92.21 ± 10.91	112.56 ± 8.41	
G-CSF	F	23.69 ± 1.61	0.6 ± 0.12	17.29 ± 2.14	19.66 ± 1.62	3 vs 6, 3 vs 10, 3 vs 15, 6 vs 10, 6 vs 15
	M	33.17 ± 3.90	0.78 ± 0.17	17.12 ± 0.96	19.70 ± 1.97	
GM-CSF	F	147.28 ± 10.53	44.48 ± 2.71	142.02 ± 21.06	171.89 ± 5.43	3 vs 6, 3 vs 15, 6 vs 10, 6 vs 15, 10 vs 15
	M	191.46 ± 19.15	43.21 ± 5.00	133.93 ± 9.02	162.72 ± 14.26	
M-CSF	F	12.89 ± 1.16	1.95 ± 0.26	11.83 ± 0.57	13.44 ± 1.56	3 vs 6, 3 vs 15, 6 vs 10, 6 vs 15
	M	16.76 ± 2.10	3.13 ± 0.31	11.25 ± 1.11	13.62 ± 1.59	
CXCL1	F	57.18 ± 6.20	14.93 ± 0.87	59.46 ± 4.25	66.56 ± 6.79	3 vs 6, 6 vs 10, 6 vs 15, 10 vs 15
	M	56.66 ± 8.30	14.16 ± 1.46	51.42 ± 4.42	65.43 ± 4.30	
CCL3	F	15.98 ± 1.23	3.72 ± 0.48	15.65 ± 0.65	18.98 ± 1.94	3 vs 6, 3 vs 15, 6 vs 10, 6 vs 15, 10 vs 15
	M	17.58 ± 2.81	4.20 ± 0.63	15.01 ± 0.86	18.94 ± 1.97	
CCL20	F	10.59 ± 0.43	1.13 ± 0.08	9.96 ± 0.76	11.53 ± 0.76	3 vs 6, 3 vs 10, 6 vs 10, 6 vs 15, 10 vs 15
	M	11.06 ± 0.54	1.18 ± 0.23	9.63 ± 0.37	11.43 ± 1.01	

CCL5	F	69.13 ± 2.43	15.45 ± 1.35	54.57 ± 6.34	65. 42 ± 3.72	3 vs 6, 3 vs 10, 6 vs 10, 6 vs 15, 10 vs 15
	M	76.04 ± 13.69	16.84 ± 1.82	52.96 ± 3.97	65. 98 ± 4.88	
CCL2	F	168.57 ± 11.23	61.23 ± 3.98	150.16 ± 15.29	193.72 ± 14.53	3 vs 6, 3 vs 15, 6 vs 10, 6 vs 15, 10 vs 15
	M	214.14 ± 21.54	64.77 ± 3.49	146.41 ± 8.61	191.91 ± 19.34	
VEGF	F	484.09 ± 34.84	112.30 ± 18.99	371.21 ± 20.18	501.11 ± 48.80	3 vs 6, 3 vs 10, 6 vs 10, 6 vs 15, 10 vs 15
	M	502.50 ± 68.97	123.39 ± 11.47	385.81 ± 29.92	502.11 ± 27.94	

*Significant differences and direction determined by one-way ANOVA.
